# The Roles of General Health and COVID-19 Proximity in Contact Tracing App Usage: Cross-sectional Survey Study

**DOI:** 10.2196/27892

**Published:** 2021-08-18

**Authors:** Dirk Witteveen, Pablo de Pedraza

**Affiliations:** 1 Nuffield College University of Oxford Oxford United Kingdom; 2 European Commission, DG Joint Research Centre Directorate I – Competences Unit I.1 - Monitoring, Indicators and Impact Evaluation Ispra (VA) Italy

**Keywords:** COVID-19, contact tracing, socioeconomic factors, labor market status, privacy, data sharing, pandemic, mobile health, public health, smartphone, mobile phone

## Abstract

**Background:**

Contact tracing apps are considered useful means to monitor SARS-CoV-2 infections during the off-peak stages of the COVID-19 pandemic. Their effectiveness is, however, dependent on the uptake of such COVID-19 apps.

**Objective:**

We examined the role of individuals’ general health status in their willingness to use a COVID-19 tracing app as well as the roles of socioeconomic characteristics and COVID-19 proximity.

**Methods:**

We drew data from the WageIndicator Foundation Living and Working in Coronavirus Times survey. The survey collected data on labor market status as well as the potential confounders of the relationship between general health and COVID-19 tracing app usage, such as sociodemographics and regular smartphone usage data. The survey also contained information that allowed us to examine the role of COVID-19 proximity, such as whether an individual has contracted SARS-CoV-2, whether an individual has family members and colleagues with COVID-19, and whether an individual exhibits COVID-19 pandemic–induced depressive and anxiety symptoms. We selected data that were collected in Spain, Italy, Germany, and the Netherlands from individuals aged between 18 and 70 years (N=4504). Logistic regressions were used to measure individuals’ willingness to use a COVID-19 tracing app.

**Results:**

We found that the influence that socioeconomic factors have on COVID-19 tracing app usage varied dramatically between the four countries, although individuals experiencing forms of not being employed (ie, recent job loss and inactivity) consistently had a lower willingness to use a contact tracing app (effect size: 24.6%) compared to that of employees (effect size: 33.4%; *P*<.001). Among the selected COVID-19 proximity indicators, having a close family member with SARS-CoV-2 infection was associated with higher contact tracing app usage (effect size: 36.3% vs 27.1%; *P*<.001). After accounting for these proximity factors and the country-based variations therein, we found that having a poorer general health status was significantly associated with a much higher likelihood of contact tracing app usage; compared to a self-reported “very good” health status (estimated probability of contact tracing app use: 29.6%), the “good” (estimated probability: +4.6%; 95% CI 1.2%-8.1%) and “fair or bad” (estimated probability: +6.3%; 95% CI 2.3%-10.3%) health statuses were associated with a markedly higher willingness to use a COVID-19 tracing app.

**Conclusions:**

Current public health policies aim to promote the use of smartphone-based contact tracing apps during the off-peak periods of the COVID-19 pandemic. Campaigns that emphasize the health benefits of COVID-19 tracing apps may contribute the most to the uptake of such apps. Public health campaigns that rely on digital platforms would also benefit from seriously considering the country-specific distribution of privacy concerns.

## Introduction

Over the course of 2020, governments have adopted a range of strategies to reduce the spread of COVID-19—an infectious disease that resulted in a pandemic—while trying to keep their economies afloat. Since mobility restrictions were gradually lifted during the last phase of the first pandemic wave in Europe, contact tracing has been considered to be an effective method for disease control, particularly for preventing disease transmission via contagious individuals who are not (yet) symptomatic [1-3]. Several governments have rolled out a version of a COVID-19 contact tracing app to help identify individuals who have been in close physical contact with an infected individual. However, as contact tracing apps inevitably rely on the collection of personal health data and mobility data, privacy concerns have been raised among the public [4].

In Europe, where participation in contact tracing via smartphone apps is voluntary, the effectiveness of contact tracing is dependent on the uptake of such apps. One study showed that in order to successfully suppress virus transmission during the peak of an outbreak in a hypothetical city with 1 million inhabitants, about 80% of all smartphone users or 56% of the population aged under 70 years would have to install the contact tracer [5]. Further, by modeling data from Washington State, researchers found that over the course of 300 days in 2020, infections and deaths could be reduced by 8% and 6%, respectively, if only 15% of the population were to participate in digital contact tracing [6]. Other researchers have also found that app-based tracing remains a more effective system than conventional contact tracing if coverage exceeds 20% [7]. Thus, although COVID-19 tracing apps are relatively ineffective during pandemic spikes, they can still help to slow the spread of SARS-COV-2 in subsequent periods, even though these apps have relatively low coverage.

This study concentrated on the association between individuals’ general health status and their willingness to use a COVID-19 tracing app across several European countries and focused on Spain, Italy, Germany, and the Netherlands. These attitudes were measured in the fall of 2020, which was when the daily number of new cases was increasing (ie, the “second wave”). We ask the following question: are poorer health statuses associated with a higher willingness to share personal information in COVID-19 tracing apps? This dynamic could occur if individuals prioritize personal or public health concerns over possible data privacy concerns. Such health risk calculations tend to operate differently for individuals who perceive themselves to be more vulnerable. The higher sense of danger among at-risk groups is often found to positively correlate with distress [8]. Furthermore, research has shown that individuals’ level of engagement with disease prevention behavior increases as soon as they are able to translate an abstract societal risk into a likelihood of experiencing a disease’s most severe consequences [9,10].

We also concentrated on the moderating role of COVID-19 proximity in the relationship between general health status and the willingness to use a contact tracing app. This is because risk perceptions are known to be partially influenced by the experiences of other individuals in one’s social circle, such as family, friends, and colleagues [10,11]. Recent studies have also suggested that individuals’ risk behaviors are rather susceptible to information treatments about COVID-19 during the pandemic. For instance, learning about the severe symptoms of COVID-19 positively influences a range of protective behaviors [12] and results in individuals being less accepting of the incautious behavior of others [13]. In other words, first-hand physical and psychological experiences and observations of nearby people being affected by COVID-19 are likely to impact individuals’ attitudes and risk behaviors. Hence, we examined the relationship between a set of COVID-19 proximity indicators and individuals’ willingness to install and use a contact tracing app. We used indicators such as being tested for COVID-19, having a close family member or colleague with COVID-19, and self-reporting depression and anxiety symptoms resulting from the pandemic.

We also addressed the role that individuals’ socioeconomic characteristics have as covariates of COVID-19 tracing app support. Such characteristics included gender, migration status, age, household status, and labor market status. It is important to account for these factors because of their expected relationship with the dependent variable (COVID-19 tracing app usage). As contact tracing systems are being rapidly rolled out by current administrations, skepticism toward COVID-19 tracing apps may be rooted in general distrust toward the government, which is why the selected sociodemographics served as necessary control variables [14]. Sociodemographic factors are also predictive of general smartphone app usage and COVID-19 tracing app installation, as shown in recent studies [15]. Furthermore, it is important to account for possible general health effect heterogeneity across the aforementioned individual-level socioeconomic characteristics [16], which could also vary across European countries [15].

In sum, we expected to find significant self-reported general health status gradients in individuals’ willingness to use a COVID-19 tracing app that are dependent on a range of socioeconomic characteristics. This relationship could be mediated by country-specific associations between socioeconomic attributes and COVID-19 tracing app support. We also hypothesized that as observable pandemic-related health risks increase for individuals, their willingness to use a COVID-19 tracing app also increases.

## Methods

### Data

Observational data were drawn from the WageIndicator Living and Working in Coronavirus Times (LWCV) survey, which was filled out by web respondents between week 42 and week 49 of 2020 [17]. Respondents provided consent for their data be used in scientific research and did not receive financial compensation for their participation in the survey. All individual-level data were anonymized by WageIndicator prior to their use by academic researchers. The data set used and the analyses conducted did not contain identifiable information.

We selected respondents aged between 18 and 70 years (N=4504) from Spain (n=1936), Italy (n=562), Germany (n=1294), and the Netherlands (n=712). This was because adults aged up to 70 years have relatively large social networks and stronger connections to the labor market (eg, coworkers). Contact tracing is also believed to be the most effective when it is performed with this population [5]. The LWCV survey collects data about family structure, COVID-19 testing, self-perceived health status, and depressive and anxiety symptoms. It also contains a series of questions about individuals’ willingness to use a COVID-19 tracing app as well as data on relevant confounders, such as general smartphone and app usage. Multimedia Appendix 1 contains the sections of the questionnaire that were used for this study.

Voluntary web surveys have become common data collection tools during the pandemic. A range of policy-relevant studies that documented the initial impact that COVID-19 has on health, work, personal, and family situations relied on data from voluntary web surveys [18-21]. Two important advantages of this data collection method are that sampling is continuous and that questionnaires can be adjusted to rapidly changing situations, such as the 2020 COVID-19 pandemic. A significant drawback of voluntary web surveys is that the samples are not representative of the full population (ie, individuals who use and do not use web-based platforms). The results of such surveys therefore have to be interpreted with caution. The application of poststratification techniques can help to partly correct the bias resulting from self-selection and underrepresentation [22].

The WageIndicator Foundation is a global research organization that relies on a long-standing survey of workforces across 150 countries. The WageIndicator Foundation website receives millions of visitors annually. The WageIndicator Foundation has produced reliable estimates of mental health, data on subjective feelings such as well-being and insecurity, and web survey weighting techniques for balancing selectivity bias [23-25]. During the COVID-19 pandemic, it has enabled the exploration of mental health, anxiety, and life satisfaction determinants [26,27]. In Multimedia Appendix 2, we benchmark the LWCV study samples against those of the European Social Survey based on key sociodemographics; relatively comparable sample distributions across age and respondents’ highest education level are displayed. However, the LWCV study samples contained slightly more individuals from the 30- to 54-year age groups than those in the general population, with the exception of Spain’s population (more individuals from the ≥55-year age group). Multimedia Appendix 3 indicates that including European Social Survey–based weights led to same substantive conclusions. In accordance with recent studies that used LWCV survey data [26,27], we report unweighted estimates for the main findings. Multimedia Appendix 4 documents the model statistics and model specification checks.

### Measures

Data on COVID-19 tracing app support were derived by asking whether a respondent was willing to share both their health status and geographical location on a COVID-19 tracing app (yes vs no or do not know). The key independent variable was self-reported general health status, which was based on the following question: “How would you rate your overall health?” Respondents answered with “very good” (832/4504, 18.5%), “good” (2409/4504, 53.5%), “fair” (1082/4504, 24%), “bad” (153/4504, 3.4%), and “very bad” (28/4504, 0.6%). We merged the smaller categories—the “bad” and “very bad” categories—with the “fair” health status to aid with interpretation and used the “very good” category as the reference. Indicators of COVID-19 proximity were measured with questions on whether a close colleague or a family member has ever contracted COVID-19, one’s own COVID-19 test-taking status and their results (none, positive, negative, and awaiting result), and self-reported COVID-19 pandemic–induced depression symptoms (5-point Likert scale) and COVID-19 pandemic–induced anxiety symptoms (5-point Likert scale). Socioeconomic variables included gender, age (age group), migration background (dichotomous), partnership status (whether partners are present in the household), whether children aged under 18 years were present in the household, the highest education level obtained (low, medium, and high), urbanicity (3 categories), labor market position (employee, freelance, self-employed, inactive, and other), and how labor market position has been affected by the COVID-19 pandemic in terms of job loss and income reduction. All models accounted for the timing of the survey (week number). Multimedia Appendix 5 depicts the correlation matrix.

### Estimation

In order to gain a thorough understanding of the critical structural pathways for contact tracing app uptake and its potential country-based variation, we first estimated the marginal effects of the socioeconomic factors and COVID-19 proximity indicators by using 2 separate series of logit models. We present the results of the bivariate models (independent variables and outcome only) and multivariate models (all independent variables combined). These analyses also included an overall model with country-fixed effects, which allowed us to account for dynamics that are altogether country specific (eg, debates on general data privacy and its consequences for people’s trust in governments). Aside from learning about the relevance of these covariates for social and health policies, they also informed us about how the relationship between general health status and contact tracing app usage should be modeled. Two-sided significance tests (α=.05) were performed for all analyses.

We also estimated COVID-19 tracing app support (*Y_prob_*) based on the general health status indicator (*H*) in nested models; socioeconomic variables and COVID-19 proximity variables were added in separate steps (equation 1). The use of nested models allowed us to examine the mechanism for explaining how general health status is related to the level of contact tracing app support. The baseline model only contained country-fixed effects (*F*) and a control for survey week (*W*). In a second series of models, we added the socioeconomic matrix (***D***) and a variable matrix for respondents’ regular smartphone usage (***P***), that is, the ownership of other apps that collect health and geographic location data (dichotomous) and the total number of phone apps. The third series of models were further adjusted for the COVID-19 proximity indicators (***C***). We calculated average marginal effects to aid our interpretation of the coefficients, as per social science conventions [28]. In Multimedia Appendix 6, we replicate the key results by using country random intercepts, which present the same quantitative results as those of the reported country-fixed effects models. Equation 1 is as follows:

Y_prob_ = β_0_ + H_i_β_1_ + F_i_γ + D_i_ω + C_i_ϕ + P_i_δ + ε_i_ **(1)**

## Results

### Descriptive Statistics

Table 1 presents the number and proportion of respondents and the average proportion of respondents who support a COVID-19 tracing app for each of the independent variables. It should be noted that both of the Southern European countries have a much higher average proportion of respondents who support contact tracing apps (Italy: 282/562, 50.2%; Spain: 716/1936, 37%). Both Germany (209/1294, 16.2%) and the Netherlands (127/712, 17.8%) display distinctly lower levels of support for a COVID-19 tracing app.

**Table 1:** Descriptive statistics for COVID-19 tracing app usage in Spain, Italy, Germany, and the Netherlands. Data are from weeks 42 through 49 (year: 2020; N=4504).

Variables				Respondents, n (proportion)		Proportion of respondents who support COVID-19 tracing apps
**Key independent variable**						
	**Health status**					
		Strong	832 (0.185)		0.209	
		Good	2409 (0.535)		0.310	
		Fair or bad	1263 (0.280)		0.327	
**Independent variables**						
	**Gender**					
		Woman	1373 (0.305)		0.268	
		Man	3131 (0.695)		0.309	
	**Migration background**					
		Native-born	4323 (0.960)		0.298	
		Foreign-born	181 (0.040)		0.249	
	**Age group (years)**					
		18-29	667 (0.148)		0.237	
		30-44	1489 (0.331)		0.320	
		45-54	1286 (0.286)		0.297	
		55-70	1062 (0.236)		0.298	
	**Partnership status**					
		No partner	1617 (0.359)		0.276	
		Partner in household	2887 (0.641)		0.307	
	**Children (in the household)**					
		No children	2679 (0.595)		0.299	
		Children	1825 (0.405)		0.292	
	**Highest education level**					
		Low	933 (0.207)		0.251	
		Medium	1625 (0.361)		0.260	
		High	1946 (0.432)		0.348	
	**Labor market position**					
		Employee	2419 (0.537)		0.324	
		Freelance	189 (0.042)		0.307	
		Self-employed with employees	59 (0.013)		0.203	
		Other employment	151 (0.034)		0.311	
		Job loss and income reduction due to the COVID-19 pandemic	861 (0.191)		0.253	
		Inactive	825 (0.183)		0.261	
	**Urbanicity**					
		City or metropole	2454 (0.545)		0.313	
		Small city or town	1297 (0.288)		0.282	
		Village or rural	753 (0.167)		0.264	
	**COVID-19 pandemic–induced depression symptoms**					
		Disagree	1773 (0.394)		0.267	
		Neutral	1084 (0.241)		0.318	
		Agree	1647 (0.366)		0.313	
	**COVID-19 pandemic–induced anxiety symptoms**					
		Disagree	1415 (0.314)		0.246	
		Neutral	1090 (0.242)		0.255	
		Agree	1999 (0.444)		0.354	
	**COVID-19 test**					
		No	2976 (0.661)		0.269	
		Yes, positive	139 (0.031)		0.381	
		Yes, awaiting result	20 (0.004)		0.400	
		Yes, negative	1369 (0.304)		0.344	
	**Close colleague with COVID-19**					
		No	2060 (0.457)		0.265	
		Yes	1242 (0.276)		0.368	
		Do not know or N/A^a^	1202 (0.267)		0.285	
	**Family member with COVID-19**					
		No	3200 (0.710)		0.263	
		Yes	1170 (0.260)		0.387	
		Do not know or N/A	134 (0.030)		0.291	

^a^N/A: not applicable.

### Socioeconomic Factors

Table 2 presents the marginal effects that socioeconomic factors had on the willingness to use a COVID-19 tracing app among the full sample and the four countries separately. The bivariate associations (marginal effect sizes) in the *Country-fixed effects* column suggest that older individuals (aged 45-54 years: 30.9%; aged 55-70 years: 31.5%; *P*<.001) are significantly more willing to use a COVID-19 tracing app than young adults (about 22.5%). Partnered individuals who also live in the same household are also more likely to use a contact tracing app than nonpartnered individuals, as indicated by the 4% marginal effects gap. Individuals with medium (effect size: 30.1%) and high (effect size: 31.6%) levels of education had a significantly higher willingness to use a COVID-19 tracing app compared to that of individuals with low levels of education (effect size: 24.7%; *P*<.001). Furthermore, compared to employees (effect size: 33.4%), individuals who are not active in the labor force (effect size: 24.6%; *P*<.001) and those who lost their job or income during the COVID-19 pandemic (effect size: 25.2%; *P*<.001) are significantly less likely to use a contact tracing app. These independent socioeconomic variables remained statistically significant in the multivariate model. Notably, gender, migration background, and urbanicity are not associated with the probability of using a COVID-19 tracing app.

The columns of Table 2 present the bivariate and multivariate model results for each of the four countries. The main finding from these models is the striking cross-national variation in the relationship between socioeconomic variables and COVID-19 tracing app usage. The impact that respondents from Spain had on the full-sample results appears to be substantial, as the country largely exhibits the same socioeconomic relationships in terms of the significance levels and magnitudes reported by the multivariate models. However, in Italy, the most important socioeconomic factors are labor market status and urbanicity. Relative to employees (effect size: 53.5%), respondents who experienced a recent job loss (effect size: 40.2%; *P*=.03) and inactive respondents (effect size: 32.2%; *P*=.04) had significantly lower probabilities of being willing to use a COVID-19 tracing app. These substantial differences were derived from multivariate models that accounted for important confounders, such as age and household status.

**Table 2:** The marginal effects that sociodemographic factors have on respondents’ willingness to use a COVID-19 tracing app. Data are from weeks 42 through 49 (2020).

Sociodemographic factors			Country-fixed effects				Spain			Italy				Germany				The Netherlands		
			Bivariate model		Multivariate model		Bivariate model		Multivariate model	Bivariate model		Multivariate model		Bivariate model		Multivariate model		Bivariate model		Multivariate model
**Gender**																				
	Woman (referent)	0.302		0.304		0.376		0.379		0.509	0.512		0.176		0.175		0.170		0.173	
	Man	0.282		0.279		0.356		0.348		0.477	0.467		0.135		0.136		0.197		0.191	
**Migration background**																				
	Native-born (referent)	0.298		0.298		0.372		0.373		0.504	0.503		0.165		0.165		0.178		0.179	
	Foreign-born	0.248		0.244		0.327		0.321		0.375	0.396		0.091		0.084		0.214		0.166	
**Age group (years)**																				
	18-29 (referent)	0.225		0.227		0.231		0.235		0.466	0.494		0.213		0.197		0.165		0.158	
	30-44	0.306^a^		0.300^a^		0.381^a^		0.374^a^		0.536	0.527		0.184		0.189		0.122		0.115	
	45-54	0.309^a^		0.314^a^		0.426^a^		0.428^a^		0.439	0.448		0.133^a^		0.145		0.225		0.232	
	55-70	0.315^a^		0.316^a^		0.412^a^		0.418^a^		0.537	0.528		0.152		0.137		0.184		0.191	
**Partnership**																				
	No partner (referent)	0.271		0.277		0.333		0.357		0.462	0.474		0.153		0.133		0.167		0.173	
	Partner in household	0.311^a^		0.307^a^		0.392^a^		0.377		0.525	0.518		0.166		0.179^a^		0.184		0.181	
**Children (in household)**																				
	No children (referent)	0.297		0.310		0.355		0.380		0.503	0.508		0.185		0.195		0.181		0.189	
	Children	0.295		0.277		0.390		0.357		0.500	0.489		0.128^a^		0.118^a^		0.175		0.164	
**Highest education level**																				
	Low (referent)	0.247		0.246		0.330		0.326		—^b^	—		0.104		0.105		0.113		0.113	
	Medium	0.301^a^		0.304^a^		0.366		0.368		0.507	0.517		0.172^a^		0.171^a^		0.173		0.173	
	High	0.316^a^		0.314^a^		0.396^a^		0.398^a^		0.499	0.493		0.171^a^		0.171^a^		0.227^a^		0.227^a^	
**Labor market position**																				
	Employee (referent)	0.334		0.327		0.434		0.411		0.536	0.535		0.181		0.181		0.186		0.183	
	Freelance	0.277		0.277		0.333		0.347		0.500	0.509		0.125		0.113		0.214		0.212	
	Self-employed with employees	0.246		0.237		0.316		0.305		0.333	0.316		0.094		0.096		0.400		0.354	
	Other employment	0.294		0.298		0.372		0.382		0.429	0.409		0.241		0.228		0.095		0.107	
	Job loss and income reduction due to the COVID-19 pandemic	0.252^a^		0.249^a^		0.336^a^		0.327^a^		0.397^a^	0.402^a^		0.100^a^		0.101^a^		0.229		0.232	
	Inactive	0.246^a^		0.266^a^		0.311^a^		0.345^a^		0.321^a^	0.322^a^		0.188		0.189		0.068^a^		0.078^a^	
**Urbanicity**																				
	City or metropole (referent)	0.296		0.294		0.371		0.367		0.458	0.459		0.176		0.176		0.206		0.201	
	Small city or town	0.293		0.295		0.361		0.366		0.558^a^	0.560^a^		0.146		0.147		0.170		0.172	
	Village or rural	0.303		0.306		0.383		0.390		0.727^a^	0.712^a^		0.140		0.140		0.163		0.163	

^a^Significant at the *P*<.05 level (two-tailed tests).

^b^Not available.

For Germany and the Netherlands, where COVID-19 tracing app support is, on average, much lower than that in the two Southern European countries, educational level and labor market position were the only statistically significant independent variables of the willingness to use a COVID-19 tracing app in the multivariate models. Holding higher education credentials in Germany and the Netherlands yielded a 6.6% higher marginal effect (Germany: *P*=.04) and an 11.4% higher marginal effect (the Netherlands: *P*=.01), respectively, on COVID-19 tracing app support compared to those for holding lower education credentials (reference group). In Germany, individuals who recently experienced a job loss or income loss are significantly less likely to use a contact tracing app (effect size: 10.1%; *P*=.01) than employees (effect size: 18.1%). In the Netherlands, the marginal effect for support for a COVID-19 tracing app is only 7.8% (*P*=.04) for those who remained inactive during the pandemic, regardless of other socioeconomic factors. Finally, in Germany, where age remained a nonsignificant independent variable, the presence of a partner (*P*=.04) and children (*P*<.001) in the household are positively associated with respondents’ willingness to use a contact tracing app.

### COVID-19 Proximity

We also examined key indicators of COVID-19 proximity. Table 3 presents the marginal effects that these indicators had on the willingness to use a COVID-19 tracing app. As shown in the *Country-fixed effects* column (the combined sample), all COVID-19 proximity factors had significant positive associations with respondents’ support for a COVID-19 tracing app. Specifically, having (potentially) contracted COVID-19 was suggestive of a higher willingness to use a COVID-19 tracing app, as indicated by the substantially higher marginal effects of being tested (positive: 35.7%; negative: 33%; awaiting results: 41.2%). However, these marginal effects ceased to be statistically significantly different from those of the reference group (no test: 27.6%) in the multivariate models. The multivariate model further indicated that reporting anxiety symptoms (the 33.2% marginal effect of the “agree” response vis-à-vis the 27.1% marginal effect of the neutral response; *P*<.001) was significantly associated with contact tracing app support. Similarly, having a family member (effect size: 35.2%; *P*<.001) or colleague (effect size: 34.9%; *P*<.001) who has ever contracted COVID-19 was also associated with greater contact tracing app support compared to not having such a family member (effect size: 27.4%) or colleague (effect size: 28.4%).

Similar to socioeconomic factors, the relationship between COVID-19 proximity and the willingness to use a COVID-19 tracing app appears to vary across the four countries studied. For instance, having no anxiety symptoms had a positive effect on COVID-19 tracing app support in Germany. Furthermore, having a colleague who tested positive for COVID-19 was associated with a higher likelihood of using a contact tracing app overall, but this was not the case in the Netherlands. In fact, having a family member who has ever contracted COVID-19 was the only indicator that yielded a significant positive marginal effect on contact tracing support across all countries. This implies that, in addition to including country-fixed effects and controls for sociodemographics and COVID-19 proximity in models, modeling the association between general health and COVID-19 tracing app support required us to ensure that the controls interact with the country dummies to account for heterogeneity.

**Table 3:** Marginal effects that COVID-19 proximity indicators had on the willingness to use a COVID-19 tracing app. Data are from weeks 42 through 49 (2020).

COVID-19 proximity indicators			Country-fixed effects				Spain				Italy				Germany				The Netherlands		
			Bivariate model		Multivariate model		Bivariate model		Multivariate model		Bivariate model		Multivariate model		Bivariate model		Multivariate model		Bivariate model		Multivariate model
**COVID-19 pandemic–induced depression symptoms**																					
	Disagree	0.291		0.298		0.380		0.403		0.523		0.539		0.131		0.135		0.186		0.206	
	Neutral (referent)	0.325		0.321		0.430		0.417		0.543		0.520		0.166		0.165		0.166		0.156	
	Agree	0.283^a^		0.280		0.335^a^		0.329^a^		0.456		0.453		0.232^a^		0.218		0.178		0.165	
**COVID-19 pandemic–induced anxiety symptoms**																					
	Disagree	0.256		0.261		0.267^a^		0.263^a^		0.425		0.428		0.250^a^		0.230^a^		0.117		0.113^a^	
	Neutral (referent)	0.274		0.271		0.369		0.362		0.413		0.414		0.134		0.141		0.171		0.178	
	Agree	0.334^a^		0.332^a^		0.431^a^		0.437^a^		0.570^a^		0.568^a^		0.115		0.119		0.268^a^		0.272^a^	
**COVID-19 test**																					
	No (referent)	0.276		0.286		0.343		0.356		0.515		0.524		0.127		0.134		0.184		0.197	
	Yes, positive	0.357^a^		0.316		0.451		0.433		0.526		0.499		0.200		0.173		0.235		0.202	
	Yes, awaiting result	0.412		0.411		0.571		0.608		0.333		0.289		0.333		0.250		—^b^		—	
	Yes, negative	0.330^a^		0.313		0.408^a^		0.385		0.475		0.461		0.260^a^		0.235^a^		0.159		0.140	
**Close colleague with COVID-19**																					
	No (referent)	0.277		0.284		0.363		0.374		0.454		0.464		0.132		0.144		0.192		0.209	
	Yes	0.364^a^		0.349^a^		0.448^a^		0.420		0.550^a^		0.543		0.262^a^		0.219^a^		0.208		0.197	
**Family member with COVID-19**																					
	No (referent)	0.271		0.274		0.344		0.349		0.464		0.470		0.144		0.148		0.157		0.158	
	Yes	0.363^a^		0.352^a^		0.430^a^		0.415^a^		0.592^a^		0.582^a^		0.265^a^		0.222^a^		0.225^a^		0.219^a^	

^a^Significant at the *P*<.05 level (two-tailed tests).

^b^Not available.

### General Health Status

**Figure 1:**
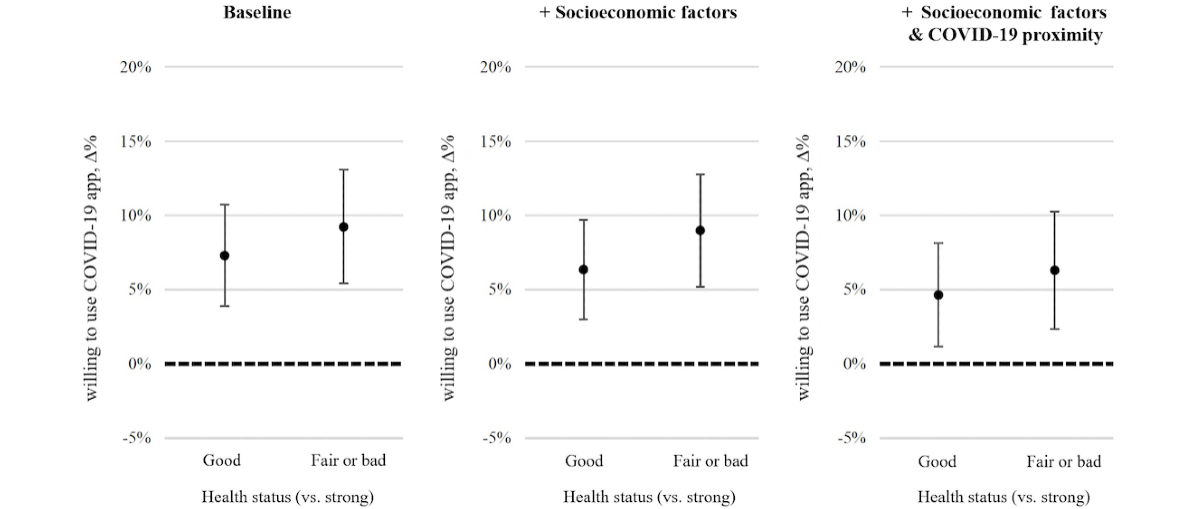
The marginal effects that poorer health statuses have on the willingness to use a COVID-19 tracing app. Country-fixed effects (Spain, Italy, Germany, and the Netherlands) are applied. Data are from weeks 42 through 49 (2020). The plots show the marginal effects of poorer health statuses, and the “strong” health status was used as the reference category. The baseline model only controlled for survey week. The error bars represent 95% CIs.

We illustrate the association between general health status and the willingness to use a COVID-19 tracing app in Figure 1. For the leftmost graph of Figure 1, we estimated a baseline model that adjusts for timing (survey week) and country-fixed effects. The graph suggests that both the “good” (estimated probability of contact tracing app use: +7.3%; *P*<.001) and “fair or bad” (estimated probability of contact tracing app use: +9.2%; *P*<.001) health statuses are positively associated with COVID-19 tracing app usage when compared to the reference category (the “strong” health status). The negative association between self-reported general health and COVID-19 tracing app usage persisted in terms of significance and magnitude when we also controlled for socioeconomic variables. However, as shown in the rightmost graph of Figure 1, the effect sizes of the poorer general health statuses were reduced by about one-third when adding the COVID-19 proximity variables; the estimated probability of contact tracing app use based on having a “good” health status and “fair or bad” health status increased by 4.6% (*P*=.01) and 6.3% (*P*=.002), respectively. This attenuation suggests that the relationship between general health status and support for a contact tracing app partially operates through recent personal health scares or forms of stress that are related to direct experiences with the COVID-19 pandemic (ie, exhibiting depressive or anxiety symptoms and a diagnosis of COVID-19 among close contacts).

**Figure 2:**
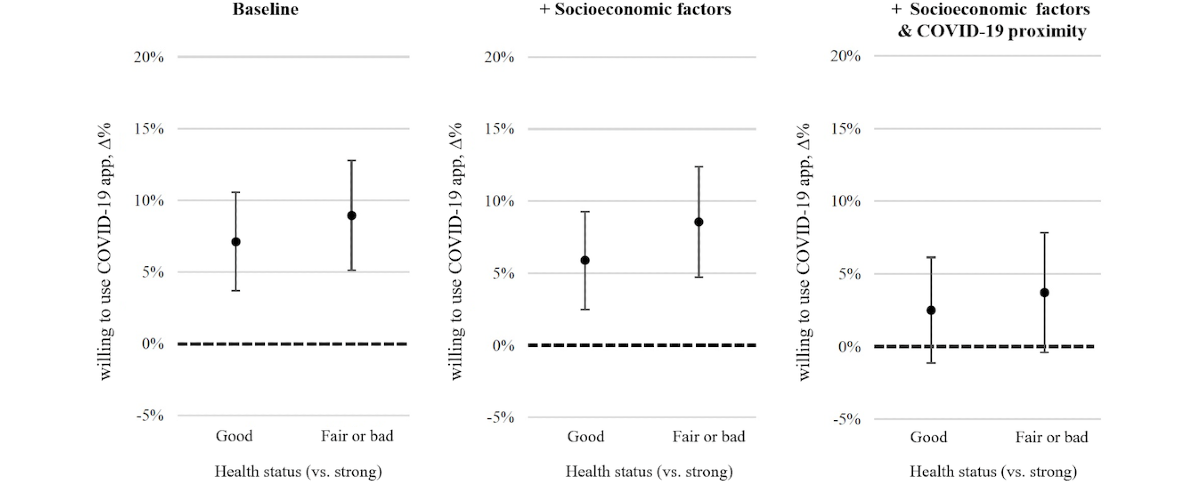
The marginal effects that poorer health statuses have on the willingness to use a COVID-19 tracing app. Country dummy interactions (Spain, Italy, Germany, and the Netherlands) with all covariates are applied. Data are from weeks 42 through 49 (2020). The plots show the marginal effects of poorer health statuses, and the “strong” health status was used as the reference category. The baseline model only controlled for survey week, which also interacted with country dummies. The error bars represent 95% CIs.

In order to correct the estimates for country heterogeneity in the relationship between the socioeconomic and COVID-19 proximity variables and the outcome variable, we analyzed the marginal effects that poorer general health has on COVID-19 tracing app support while adjusting for the interactions between all covariates and the country dummies (Figure 2). As the point estimates in the baseline and socioeconomic models presented in Figure 2 are nearly identical to those in Figure 1, we conclude that neither timing or socioeconomic heterogeneity influences the identified relationship between general health status and the willingness to use a COVID-19 tracing app across the four European countries. However, adjusting for the country heterogeneity in the COVID-19 proximity factors yielded evident null effects (rightmost graph in Figure 2). Hence, the association between (poorer) general health status and COVID-19 tracing app support varies across the four countries, but this is likely due to the varying degree to which the COVID-19 proximity measures are associated with COVID-19 tracing app support.

## Discussion

### Principal Findings

An important component of public health policy with regard to the spread of COVID-19 is the possibility of using mobile phone–based contact tracing in response to a positive COVID-19 test [2,3]. This is particularly relevant outside of major peaks in SARS-CoV-2 infection rates and lockdowns because it could help with avoiding rapid and uncontrollable disease transmission within communities [4]. COVID-19 tracing apps have been introduced in several countries. The extent to which these contact tracing apps can have a positive effect on public health (ie, reduce the chance of rapid outbreaks) is dependent on their uptake [5-7]. We found considerable country-based variation in the willingness to use a COVID-19 tracing app, which ranged from 16.2% (209/1294) in Germany to 50.2% (282/562) in Italy. In addition, we found evidence indicating that several socioeconomic and demographic factors are associated with the willingness to use a COVID-19 contact tracing app. Such evidence was also found for the following COVID-19 proximity variables: exhibiting depression and anxiety symptoms, being tested, and having family members or colleagues who have ever contracted COVID-19. Importantly, based on these dynamics, we found that poorer health statuses are associated with significantly higher support for COVID-19 contact tracing apps.

We examined indicators of COVID-19 proximity because, based on exiting literature, we presumed that being confronted with the detrimental effects of the pandemic within one’s social network may trigger the motivation to install and use a COVID-19 tracing app [8-13]. Although we cannot observe changes in individuals’ attitudes or behaviors in response to COVID-19 proximity over time, our findings support this relationship. We observed some cross-national variation in the significance levels of the COVID-19 proximity indicators. Nonetheless, having a family member has ever tested positive for COVID-19 appears to the strongest and most consistent independent variable of the increased intended usage of a contact tracing app. We argue that these results are suggestive of a relationship between the social context of the consequences of the COVID-19 pandemic and individuals’ perceived risk.

Given the cross-national variation in the associations between our two sets of control variables (socioeconomic factors and COVID-19 proximity), we fitted a comprehensive model that adjusted for this heterogeneity (ie, the heterogeneity resulting from the covariates interacting with the country-fixed effects) [14-16]. The results from this model were straightforward. We found that having a poorer general health status (ie, the “fair or bad” or “good” health status vis-à-vis the “very good” health status) positively affects the willingness to use a COVID-19 tracing app, even after adjusting for socioeconomic factors, indicators of how close the pandemic came to an individual (COVID-19 proximity indicators), and regular levels of smartphone usage. Our results indicate that this relationship is moderated by COVID-19 proximity. In other words, it is plausible that the association between general health status and contact tracing app uptake is affected by how close the pandemic has come to an individual.

It is also important to note that the recent loss of one’s job or main income source during the pandemic yielded a significantly lower marginal effect on the willingness to use a contact tracing app in Spain, Italy, and Germany (differences of almost 10%), and similar results were observed for long-term inactivity in the Netherlands. We suspect that some of these associations reflect the impacts that economic security and insecurity have on sentiments regarding the pandemic or even a broader (structural) rejection of government (social) policies. These dynamics require much more in-depth research in the social science field.

### Limitations

Our analyses relied on cross-sectional data that were obtained during the COVID-19 pandemic. We conducted several robustness checks to avoid having strong selection bias in the reported marginal effects of general health status, socioeconomic characteristics, and COVID-19 proximity. Such bias is likely to result from the underrepresentation of individuals with the poorest general health conditions—a demographic group that is difficult to include in all kinds of social surveys. Nationally representative panels would be the preferred data source for future research on the relationship between health and any kind of COVID-19–related measures. This is because longitudinal data are better equipped to measure (deteriorating) health-related attrition. Furthermore, panel data are also best suited for effectively measuring the degree to which attitudes and behaviors of individuals change over time as a function of their health status or in response to the acquisition of new information. Future research may also benefit from expanding the operationalization of health risk and risk perceptions, such as those related to wearing a mask in close proximity to an individual.

### Conclusions

This paper builds upon existing evidence indicating that contact tracing apps are an important element of public health [2,3] and that their positive effect is dependent on their uptake [5-7]. We studied whether general health status and COVID-19 proximity can be linked to contact tracing app uptake. This research question was motivated by a discussion in public health literature about the necessity of effective contact tracing in combating the COVID-19 pandemic as well as research in the social science field regarding the individual-level drivers of attitudes toward contact tracing apps [8-13]. We conclude that poorer general health statuses are positively associated with the willingness to use a COVID-19 tracing app. Moreover, the extent to which one’s general health status impacts their likelihood of using COVID-19 tracing apps partially operates through the pandemic-related experiences that occur in their social circle.

To date, public debates have mainly revolved around issues regarding apps’ capacity to meet data privacy goals and legislation criteria. We suspect that the country-based variation we found in people’s willingness to use a COVID-19 tracing app reflects path-dependent societal dimensions, such as large personal data leaks in recent history or underlying distrust in the government [14]. This implies that public policies that are intended to expand the usage of digital COVID-19 contact tracing apps always have to consider country-specific societal concerns. Our study suggests that once these conditions are met, public health policies that aim to increase contact tracing app uptake would benefit from campaigns that stress these apps’ benefits for users (both physical and mental benefits), their family members, and the economy [1].

## Appendix

Multimedia Appendix 1 Survey questions.

Multimedia Appendix 2 Key demographic proportions: European Social Survey and WageIndicator LWCV study sample.

Multimedia Appendix 3 Replication with European Social Survey-derived weights.

Multimedia Appendix 4 Model statistics and specification checks.

Multimedia Appendix 5 Correlation matrix of all predictor variables.

Multimedia Appendix 6 Associations of socioeconomic factors and COVID-19 proximity with country — random intercepts.

## Abbreviations

LWCV: Living and Working in Coronavirus Times
