# Supplementary material for: The Roles of General Health and COVID-19 Proximity in Contact Tracing App Usage: Cross-sectional Survey Study
Source: JMIR Public Health Surveill. 2021 Aug 18;7(8):e27892. doi: 10.2196/27892 (PMC8382155; doi:10.2196/27892)
Supplement: Multimedia Appendix 1 [file publichealth_v7i8e27892_app1.docx]

**Supplement 1**. Survey Questions.

Link to LWCV codebook and questionnaire: <https://wageindicator.org/documents/publicationslist/publications-2020/corona-survey-codebook_20200330.pdf>

Data are available at the IZA Data Repository:

<https://datasets.iza.org/dataset/1388/living-and-working-in-coronavirus-times-survey>

Questions relevant for the study:

**Dependent variable: COVID-19 app support.**

Q: Are you willing to share geographical information and use a COVID-19 app?

1. No.
2. Yes.
3. Don’t know.

Q: Are you willing to share health information and use a COVID-19 app?

1. No.
2. Yes.
3. Don’t know.

**Key independent variable.**

Q: How would you rate your health at present?

1. Very good.
2. Good.
3. Fair.
4. Bad.
5. Very bad.

**Socioeconomic independent variables.**

Q: What is your gender?

1. Woman.
2. Man.

Q: In which country were you born? [Prefilled list of countries]

Q: When were your born? [open] Converted to age.

Q: What is the highest level of education you attained? [open] Converted to:

1. Low
2. Medium
3. High

Q: With whom do you live in the household? [multiple checks possible]

1. Spouse / partner
2. One or more children

Q: What is your employment status?

1. Employee
2. Self-employed without employees / freelance
3. Self-employed with employees
4. Paid family worker
5. Casual worker
6. Other

Q: Is your work affected by the corona virus? [multiple checks possible]

1. The workload has increased.
2. The workload has decreased.
3. I have to work from home.
4. I have lost my job.

Q: Where do you live? [country and country-dependent province, state, region, city] Converted to:

1. A large city.
2. The suburbs of a large city / metropolitan area.
3. A city (100,000 – 1 million).
4. A small city or town (10,000 – 100,000)
5. A village (less than 10,000)
6. Rural area
7. Metropolitan city.

**COVID-19 proximity independent variables.**

Q: The pandemic makes me feel depressed [1]:

1. Completely disagree.
2. Somewhat disagree.
3. Neutral.
4. Somewhat agree.
5. Completely agree.

Q: The pandemic makes me feel anxious [2]:

1. Completely disagree.
2. Somewhat disagree.
3. Neutral.
4. Somewhat agree.
5. Completely agree.

Q: Have you been tested for the coronavirus?

1. No
2. Yes, and I have been diagnosed with the coronavirus.
3. Yes, and I am waiting for the results.
4. Yes, and I do not have the virus.

Q: Have any of your family or friends been diagnosed with the coronavirus?

1. No.
2. Yes.
3. Don’t know.

Q: Have any of your colleagues at work been diagnosed with the coronavirus?

1. No.
2. Yes.
3. Don’t know.
