# Supplementary material for: The Roles of General Health and COVID-19 Proximity in Contact Tracing App Usage: Cross-sectional Survey Study
Source: JMIR Public Health Surveill. 2021 Aug 18;7(8):e27892. doi: 10.2196/27892 (PMC8382155; doi:10.2196/27892)
Supplement: Multimedia Appendix 2 [file publichealth_v7i8e27892_app2.docx]

**Supplement 2**. Key Demographic Proportions: European Social Survey and Wage Indicator Study Sample.

|  |  |  | **Spain** | | |  | **Italy** | | |  | **Germany** | | |  | **Netherlands** | | |
| --- | --- | --- | --- | --- | --- | --- | --- | --- | --- | --- | --- | --- | --- | --- | --- | --- | --- |
|  |  |  | ESS |  | WI |  | ESS |  | WI |  | ESS |  | WI |  | ESS |  | WI |
| Age | |  |  |  |  |  |  |  |  |  |  |  |  |  |  |  |  |
|  | 18-29 |  | .192 |  | .169 |  | .199 |  | .103 |  | .198 |  | .094 |  | .187 |  | .162 |
|  | 30-44 |  | .354 |  | .285 |  | .250 |  | .399 |  | .240 |  | .315 |  | .254 |  | .242 |
|  | 45-54 |  | .252 |  | .245 |  | .221 |  | .279 |  | .208 |  | .331 |  | .228 |  | .299 |
|  | 55-70 |  | .202 |  | .301 |  | .329 |  | .219 |  | .354 |  | .260 |  | .332 |  | .298 |
| Education | |  |  |  |  |  |  |  |  |  |  |  |  |  |  |  |  |
|  | ISCED 4 or lower |  | .749 |  | .778 |  | .864 |  | .893 |  | .688 |  | .747 |  | .684 |  | .665 |
|  | ISCED 5A or higher |  | .251 |  | .222 |  | .136 |  | .107 |  | .312 |  | .253 |  | .316 |  | .335 |
|  | | | | | | | | | | | | | | | | | |

*Source*. ESS = European Social Survey (2016-2018). WI = WageIndicator Survey: Living and Working in Coronavirus Times 2020 (LWCV). ISCED = International Standard Classification of Education.
