# Supplementary material for: The Roles of General Health and COVID-19 Proximity in Contact Tracing App Usage: Cross-sectional Survey Study
Source: JMIR Public Health Surveill. 2021 Aug 18;7(8):e27892. doi: 10.2196/27892 (PMC8382155; doi:10.2196/27892)
Supplement: Multimedia Appendix 3 [file publichealth_v7i8e27892_app3.docx]

**Supplement 3**. Replication with European Social Survey-derived Weights.

**Table S3a**. Marginal Effects of Socioeconomic Factors on Willingness to use COVID-19 app.

|  |  |  | **country-fixed effects** | |  | **Spain** | |  | **Italy** | |  | **Germany** | |  | **Netherlands** | |
| --- | --- | --- | --- | --- | --- | --- | --- | --- | --- | --- | --- | --- | --- | --- | --- | --- |
|  |  |  | bivariate | multivariate |  | bivariate | multivariate |  | bivariate | multivariate |  | bivariate | multivariate |  | bivariate | multivariate |
| Gender | |  |  |  |  |  |  |  |  |  |  |  |  |  |  |  |
|  | woman *(ref)* |  | .284 | .281 |  | .358 | .347 |  | .497 | .478 |  | .138 | .140 |  | .206 | .194 |
|  | man |  | .308 | .309 |  | .385 | .388 |  | .518 | .521 |  | .180 | .178 |  | .175 | .179 |
| Migration background | |  |  |  |  |  |  |  |  |  |  |  |  |  |  |  |
|  | native-born *(ref)* |  | .305 | .305 |  | .381 | .381 |  | .519 | .518 |  | .170 | .170 |  | .182 | .183 |
|  | foreign-born |  | .248 | .245 |  | .337 | .332 |  | .240 | .269 |  | .088 | .088 |  | .210 | .171 |
| Age group | |  |  |  |  |  |  |  |  |  |  |  |  |  |  |  |
|  | 18-29 *(ref)* |  | .223 | .224 |  | .240 | .243 |  | .504 | .510 |  | .208 | .196 |  | .141 | .137 |
|  | 30-44 |  | .311*** | .306*** |  | .387*** | .378*** |  | .536 | .531 |  | .194 | .201 |  | .130 | .132 |
|  | 45-54 |  | .316*** | .321*** |  | .441*** | .446*** |  | .452 | .462 |  | .135 | .144 |  | .221 | .223 |
|  | 55-70 |  | .332*** | .333*** |  | .437*** | .443*** |  | .558 | .551 |  | .158 | .145 |  | .211 | .210 |
| Partnership | |  |  |  |  |  |  |  |  |  |  |  |  |  |  |  |
|  | no partner *(ref)* |  | .278 | .287 |  | .340 | .364 |  | .515 | .518 |  | .154 | .138 |  | .167 | .178 |
|  | partner in household |  | .316* | .310 |  | .402* | .387 |  | .514 | .513 |  | .173 | .182 |  | .191 | .185 |
| Children (in household) | |  |  |  |  |  |  |  |  |  |  |  |  |  |  |  |
|  | no children *(ref)* |  | .302 | .315 |  | .365 | .392 |  | .523 | .522 |  | .182 | .193 |  | .185 | .193 |
|  | children |  | .302 | .284* |  | .398 | .361 |  | .499 | .501 |  | .143 | .132* |  | .179 | .169 |
| Highest education | |  |  |  |  |  |  |  |  |  |  |  |  |  |  |  |
|  | low *(ref)* |  | .252 | .251 |  | .328 | .325 |  | - | - |  | .108 | .109 |  | .105 | .106 |
|  | medium |  | .297* | .300* |  | .364 | .366 |  | .511 | .521 |  | .170* | .170 |  | .173 | .173 |
|  | high |  | .321*** | .319*** |  | .404** | .404** |  | .516 | .513 |  | .167* | .167 |  | .232** | .231** |
| Labor market position | |  |  |  |  |  |  |  |  |  |  |  |  |  |  |  |
|  | employee *(ref)* |  | .343 | .335 |  | .444 | .423 |  | .550 | .549 |  | .186 | .185 |  | .193 | .190 |
|  | freelance |  | .283 | .287 |  | .347 | .364 |  | .500 | .505 |  | .168 | .161 |  | .183 | .173 |
|  | self-employed /w employees |  | .236 | .229 |  | .331 | .320 |  | .419 | .418 |  | .070 | .072 |  | .361 | .312 |
|  | other employment |  | .285 | .290 |  | .373 | .385 |  | .300 | .304 |  | .225 | .222 |  | .065 | .072 |
|  | lost/decrease during COVID-19 |  | .249*** | .248*** |  | .337** | .328** |  | .381* | .380* |  | .101** | .104** |  | .222 | .232 |
|  | inactive |  | .256*** | .275** |  | .317*** | .351* |  | .406 | .421 |  | .195 | .193 |  | .072* | .081* |
| Urbanicity | |  |  |  |  |  |  |  |  |  |  |  |  |  |  |  |
|  | city or metropole *(ref)* |  | .302 | .300 |  | .379 | .374 |  | .481 | .483 |  | .173 | .174 |  | .208 | .204 |
|  | small city or town |  | .297 | .299 |  | .367 | .373 |  | .548 | .548 |  | .167 | .167 |  | .167 | .169 |
|  | village or rural |  | .314 | .318 |  | .398 | .408 |  | .737** | .718* |  | .148 | .146 |  | .184 | .184 |
|  | | | | | | | | | | | | | | | | |

*Note*. * p <.05; ** p <.01; *** p <.001 (two-tailed tests).

**Table S3b**. Marginal Effects of COVID-19 Proximity Indicators on Willingness to use COVID-19 app.

|  |  |  | **country-fixed effects** | |  | **Spain** | |  | **Italy** | |  | **Germany** | |  | **Netherlands** | |
| --- | --- | --- | --- | --- | --- | --- | --- | --- | --- | --- | --- | --- | --- | --- | --- | --- |
|  |  |  | bivariate | multivariate |  | bivariate | multivariate |  | bivariate | multivariate |  | bivariate | multivariate |  | bivariate | multivariate |
| Depression symptoms (COVID-19) | |  |  |  |  |  |  |  |  |  |  |  |  |  |  |  |
|  | disagree |  | .289* | .294 |  | .385 | .410 |  | .508 | .516 |  | .128 | .133 |  | .196 | .216 |
|  | neutral *(ref)* |  | .331 | .327 |  | .445 | .431 |  | .524 | .509 |  | .169 | .165 |  | .172 | .163 |
|  | agree |  | .297 | .294 |  | .343** | .337** |  | .515 | .517 |  | .261* | .246* |  | .174 | .160 |
| Anxiety symptoms (COVID-19) | |  |  |  |  |  |  |  |  |  |  |  |  |  |  |  |
|  | disagree |  | .267 | .273 |  | .275** | .272** |  | .467 | .479 |  | .261*** | .240** |  | .114 | .109* |
|  | neutral *(ref)* |  | .279 | .277 |  | .376 | .370 |  | .411 | .412 |  | .138 | .147 |  | .175 | .181 |
|  | agree |  | .336** | .333*** |  | .438** | .442* |  | .568* | .563* |  | .115 | .118 |  | .278* | .284* |
| COVID-19 test | |  |  |  |  |  |  |  |  |  |  |  |  |  |  |  |
|  | no *(ref)* |  | .279 | .288 |  | .347 | .360 |  | .533 | .540 |  | .129 | .135 |  | .192 | .207 |
|  | yes, positive |  | .363* | .324 |  | .444 | .424 |  | .511 | .480 |  | .299 | .254 |  | .243 | .224 |
|  | yes, awaiting result |  | .449 | .443 |  | .597 | .635 |  | .380 | .331 |  | .376 | .290 |  | - | - |
|  | yes, negative |  | .339*** | .325* |  | .423** | .401 |  | .485 | .476 |  | .264*** | .243*** |  | .155 | .137* |
| Close colleague COVID-19 | |  |  |  |  |  |  |  |  |  |  |  |  |  |  |  |
|  | no *(ref)* |  | .282 | .290 |  | .365 | .376 |  | .476 | .486 |  | .136 | .153 |  | .207 | .222 |
|  | yes |  | .368*** | .352*** |  | .460** | .432 |  | .541 | .536 |  | .268*** | .219* |  | .207 | .196 |
| Family member COVID-19 | |  |  |  |  |  |  |  |  |  |  |  |  |  |  |  |
|  | no *(ref)* |  | .274 | .278 |  | .350 | .356 |  | .466 | .470 |  | .146 | .151 |  | .161 | .162 |
|  | yes |  | .369*** | .358*** |  | .438** | .423** |  | .610** | .606* |  | .266*** | .220* |  | .226 | .220 |
|  | | | | | | | | | | | | | | | | |

*Note*. * p <.05; ** p <.01; *** p <.001 (two-tailed tests).

**Table S3c.** Marginal Effects of Poorer Health Statuses on Willingness to use COVID-19 app.

|  |  |  | **country-fixed effects** | |  | **country-interactions with covariates** | |
| --- | --- | --- | --- | --- | --- | --- | --- |
|  |  |  | ∆% | s.e. |  | ∆% | s.e. |
| baseline | |  |  |  |  |  |  |
|  | very good |  | *ref.* | |  | *ref.* | |
|  | good |  | + 7.6%*** | 1.9 |  | + 7.4%*** | 1.9 |
|  | fair-bad |  | + 9.8%*** | 2.1 |  | + 9.4%*** | 2.1 |
|  | | | | | | | |
| + socioeconomic factors | |  |  |  |  |  |  |
|  | very good |  | *ref.* | |  | *ref.* | |
|  | good |  | + 6.7%*** | 1.8 |  | + 6.3%** | 1.9 |
|  | fair-bad |  | + 9.2%*** | 2.1 |  | + 8.8%*** | 2.1 |
|  | | | | | | | |
| + socioeconomic factors & COVID-19 proximity | |  |  |  |  |  |  |
|  | very good |  | *ref.* | |  | *ref.* | |
|  | good |  | + 5.0%* | 1.9 |  | + 2.6% | 2.0 |
|  | fair-bad |  | + 6.3%** | 2.2 |  | + 3.6% | 2.3 |
|  | | | | | | | |

*Note*.* p <.05; ** p <.01; *** p <.001 (two-tailed tests).
