# Supplementary material for: The Roles of General Health and COVID-19 Proximity in Contact Tracing App Usage: Cross-sectional Survey Study
Source: JMIR Public Health Surveill. 2021 Aug 18;7(8):e27892. doi: 10.2196/27892 (PMC8382155; doi:10.2196/27892)
Supplement: Multimedia Appendix 4 [file publichealth_v7i8e27892_app4.docx]

**Supplement 4.** Model Statistics and Specification Checks.

**Table S4a.** Model Statistics Table 2 (multivariate only).

|  |  |  | **country FE** |  | **Spain** |  | **Italy** |  | **Germany** |  | **Netherlands** |
| --- | --- | --- | --- | --- | --- | --- | --- | --- | --- | --- | --- |
| Specification | |  |  |  |  |  |  |  |  |  |  |
|  | Link test (p) |  | .259 |  | .485 |  | .269 |  | .698 |  | .100 |
| Goodness-of-fit | |  |  |  |  |  |  |  |  |  |  |
|  | Pseudo R2 |  | .075 |  | .027 |  | .038 |  | .041 |  | .043 |
|  | LL Chi^2^ |  | 411 |  | 69 |  | 29 |  | 47 |  | 29 |
|  | Hosmer & Lemeshow Chi^2^ (p) |  | .231 |  | .238 |  | .183 |  | .833 |  | .534 |
|  | | | | | | | | | | | |

**Table S4b.** Model Statistics Table 3 (multivariate only).

|  |  |  | **country FE** |  | **Spain** |  | **Italy** |  | **Germany** |  | **Netherlands** |
| --- | --- | --- | --- | --- | --- | --- | --- | --- | --- | --- | --- |
| Specification | |  |  |  |  |  |  |  |  |  |  |
|  | Link test (p) |  | .563 |  | .193 |  | .865 |  | .142 |  | .888 |
| Goodness-of-fit | |  |  |  |  |  |  |  |  |  |  |
|  | Pseudo R2 |  | .080 |  | .034 |  | .041 |  | .079 |  | .062 |
|  | LL Chi^2^ |  | 437 |  | 88 |  | 32 |  | 91 |  | 41 |
|  | Hosmer & Lemeshow Chi^2^ (p) |  | .001 |  | .297 |  | .161 |  | .008 |  | .237 |
|  | | | | | | | | | | | |

**Table S4c.** Model Statistics Figures 1 and 2.

|  |  |  | **baseline**  **(Fig. 1)** |  | **+ soc.**  **(Fig. 1)** |  | **+ soc./covid (Fig. 3)** |  | **baseline**  **(Fig. 2)** |  | **+ soc.**  **(Fig. 3)** |  | **+ soc./covid (Fig. 4)** |
| --- | --- | --- | --- | --- | --- | --- | --- | --- | --- | --- | --- | --- | --- |
| Specification | |  |  |  |  |  |  |  |  |  |  |  |  |
|  | Link test (p) |  | .465 |  | .058 |  | .102 |  | .597 |  | .335 |  | .070 |
| Goodness-of-fit | |  |  |  |  |  |  |  |  |  |  |  |  |
|  | Pseudo R^2^ |  | .065 |  | .120 |  | .135 |  | .072 |  | .135 |  | .175 |
|  | LL Chi^2^ |  | 354 |  | 657 |  | 737 |  | 393 |  | 735 |  | 958 |
|  | Hosmer & Lemeshow Chi^2^ (p) |  | .005 |  | .736 |  | .752 |  | .026 |  | .338 |  | .440 |
| Multicollinearity | |  |  |  |  |  |  |  |  |  |  |  |  |
|  | VIF independent var. (health) |  | 4.21, 2.77 |  | 4.01, 2.68 |  | 3.99, 2.66 |  | 3.91, 2.52 |  | 4.16, 2.17 |  | 4.48, 3.07 |
| Information criterion | |  |  |  |  |  |  |  |  |  |  |  |  |
|  | BIC |  | 5133.2 |  | 4867.8 |  | 4820.2 |  | 5136.2 |  | 4894.0 |  | 4790.4 |
|  | | | | | | | | | | | | | |
