# Supplementary material for: The Roles of General Health and COVID-19 Proximity in Contact Tracing App Usage: Cross-sectional Survey Study
Source: JMIR Public Health Surveill. 2021 Aug 18;7(8):e27892. doi: 10.2196/27892 (PMC8382155; doi:10.2196/27892)
Supplement: Multimedia Appendix 5 [file publichealth_v7i8e27892_app5.docx]

**Supplement 5.** Correlation Matrix of All Predictor Variables.

|  | GHEAL | GEND | FBORN | AGE | PART | CHILD | EDUC | EMPL | URB | DEP | ANX | TEST | CCOL | CFAM | SWK | SHARE | NAPP |
| --- | --- | --- | --- | --- | --- | --- | --- | --- | --- | --- | --- | --- | --- | --- | --- | --- | --- |
| GHEAL | 1.000 |  |  |  |  |  |  |  |  |  |  |  |  |  |  |  |  |
| GEND | .052* | 1.000 |  |  |  |  |  |  |  |  |  |  |  |  |  |  |  |
| FBORN | -.049* | .027 | 1.000 |  |  |  |  |  |  |  |  |  |  |  |  |  |  |
| AGE | .058* | -.054* | .030* | 1.000 |  |  |  |  |  |  |  |  |  |  |  |  |  |
| PART | -.012 | .056* | .002 | .226* | 1.000 |  |  |  |  |  |  |  |  |  |  |  |  |
| CHILD | -.006 | .103* | .013 | .180* | .328* | 1.000 |  |  |  |  |  |  |  |  |  |  |  |
| EDUC | -.053* | .000 | .026 | -.062* | .017 | -.044* | 1.000 |  |  |  |  |  |  |  |  |  |  |
| EMPL | .046* | .009 | .040* | -.125* | -.110* | -.062* | -.143* | 1.000 |  |  |  |  |  |  |  |  |  |
| URB | .014 | .009 | -.010 | -.015 | .031* | .039* | -.114* | -.036* | 1.000 |  |  |  |  |  |  |  |  |
| DEP | .139* | .052* | .039 | -.162* | -.032* | -.020 | .008 | .129* | -.038* | 1.000 |  |  |  |  |  |  |  |
| ANX | .138* | .065* | -.023* | -.003 | .032* | .037* | -.004 | .007 | .003 | .153* | 1.000 |  |  |  |  |  |  |
| TEST | .072* | .028 | -.010 | -.019 | .036* | .006 | .045* | -.113* | -.059* | .033* | .040* | 1.000 |  |  |  |  |  |
| CCOL | .128* | .033* | .002 | -.110* | -.136* | -.084* | -.127* | .542* | -.026 | .117* | .033* | -.035* | 1.000 |  |  |  |  |
| CFAM | .054* | .027 | -.002 | -.058* | -.011 | -.068* | .028 | -.011 | -.011 | .039* | .029* | .081* | .111* | 1.000 |  |  |  |
| SWK | -.034* | -.004 | -.049* | .082* | .004 | -.057* | .014 | -.059* | .015 | -.086* | -.039* | .017 | -.027 | -.001 | 1.000 |  |  |
| SHARE | -.032* | -.010 | -.004 | -.097* | .018 | -.023 | .099* | -.085* | -.021 | -.003 | .012 | .076* | -.034* | .048* | .012 | 1.000 |  |
| NAPP | .030* | -.042* | .012 | -.065* | .032* | .009 | .135* | -.093* | -.059* | .074* | .019 | .105* | -.042* | .101* | -.098* | .620* | 1.000 |

*Notes*. GHEAL = general health status, GEND = gender, FBORN = foreign-born, AGE = age, PART = partner (in household), CHILD = children (in household), EDUC = highest education, EMPL = labor market position, URB = urbanicity, DEP = depressive symptoms, ANX = health anxiety, TEST = COVID-19 test (result), CCOL = close colleague COVID-19, CFAM = family member COVID-19, SWK = survey week, SHARE = usage of health and geographic location apps, NAPP = number of apps on phone.

* denotes significant correlation with α = .05.
