# Supplementary material for: The Roles of General Health and COVID-19 Proximity in Contact Tracing App Usage: Cross-sectional Survey Study
Source: JMIR Public Health Surveill. 2021 Aug 18;7(8):e27892. doi: 10.2196/27892 (PMC8382155; doi:10.2196/27892)
Supplement: Multimedia Appendix 6 [file publichealth_v7i8e27892_app6.docx]

**Supplement 6**. Associations of Socioeconomic Factors and COVID-19 Proximity with Country- Random Intercepts.

**Table S6a**. Marginal Effects of Socioeconomic Factors on Willingness to use COVID-19 app.

| **country-random intercepts** | | | |
| --- | --- | --- | --- |
|  |  | bivariate | multivariate |
| Gender |  |  |  |
| woman *(ref)* |  | .302 | .304 |
| man |  | .282 | .279 |
| Migration background |  |  |  |
| native-born *(ref)* |  | .298 | .298 |
| foreign-born |  | .248 | .244 |
| Age group |  |  |  |
| 18-29 *(ref)* |  | .225 | .227 |
| 30-44 |  | .306*** | .300*** |
| 45-54 |  | .309*** | .314*** |
| 55-70 |  | .315*** | .316*** |
| Partnership |  |  |  |
| no partner *(ref)* |  | .271 | .277 |
| partner in household |  | .311** | .307* |
| Children (in household) |  |  |  |
| no children *(ref)* |  | .297 | .310 |
| children |  | .295 | .277 |
| Highest education |  |  |  |
| low *(ref)* |  | .247 | .246 |
| medium |  | .301** | .304** |
| high |  | .316*** | .314*** |
| Labor market position |  |  |  |
| employee *(ref)* |  | .334 | .327 |
| freelance |  | .277 | .277 |
| self-employed /w employees |  | .246 | .237 |
| other employment |  | .294 | .298 |
| lost/decrease due COVID-19 |  | .252*** | .249*** |
| inactive |  | .246*** | .266*** |
| Urbanicity |  |  |  |
| city or metropole *(ref)* |  | .296 | .294 |
| small city or town |  | .293 | .295 |
| village or rural |  | .303 | .306 |

*Note*. A model with country-fixed effects yields the same qualitative results (Table 2). * p <.05; ** p <.01; *** p <.001 (two-tailed tests).

**Table S6b**. Marginal Effects of COVID-19 Proximity on Willingness to use COVID-19 app.

| **country-random intercepts** | | | |
| --- | --- | --- | --- |
|  |  | bivariate | multivariate |
| Depression symptoms (COVID-19) | | | |
| disagree |  | ·296 | ·296 |
| neutral *(ref)* |  | ·330 | ·319 |
| agree |  | ·289* | ·278 |
| Anxiety symptoms (COVID-19) |  |  |  |
| disagree |  | ·262 | ·261 |
| neutral *(ref)* |  | ·280 | ·271 |
| agree |  | ·340*** | ·332*** |
| COVID-19 tests |  |  |  |
| no |  | ·282 | ·285 |
| yes, positive |  | ·363* | ·315 |
| yes, awaiting result |  | ·417 | ·409 |
| yes, negative |  | ·336*** | ·312 |
| Close colleague COVID-19 |  |  |  |
| no *(ref)* |  | ·276 | ·283 |
| yes |  | ·362*** | ·347*** |
| Family member COVID-19 |  |  |  |
| no *(ref)* |  | ·276 | ·274 |
| yes |  | ·368*** | ·351*** |

*Note*. A model with country-fixed effects yields the same qualitative results (Table 3). * p <.05; ** p <.01; *** p <.001 (two-tailed tests).
